# Nutrition Knowledge among College of Basic Education Students in Kuwait: A Cross-Sectional Study

**DOI:** 10.1155/2021/5560714

**Published:** 2021-03-24

**Authors:** Wafaa Husain, Fatemah Ashkanani, Maryam A. Al Dwairji

**Affiliations:** ^1^Home Economics Department, College of Basic Education, The Public Authority for Applied Education and Training, Al-Ardiya, Kuwait; ^2^Food and Nutrition Administration, Ministry of Health, Kuwait City, Kuwait

## Abstract

Lack of nutrition knowledge may contribute to poor dietary practices. Schools are an ideal environment to address this issue and to start the process of nutrition education. Therefore, teachers should be equipped with adequate nutrition knowledge. This study was designed to evaluate the level of general nutrition knowledge and demographic variations in knowledge in a sample of students attending the College of Basic Education in Kuwait. A cross-sectional study was conducted using a modified and validated revised version of the General Nutrition Knowledge Questionnaire (GNKQ-R) for UK adults. Univariate and multivariate analyses were performed to determine the association between various factors and nutrition knowledge score. A total of five hundred and ninety-seven students completed the questionnaire. Most respondents (84.1%) had a poor level of nutrition knowledge using original Bloom's cutoff points. Sex, BMI, cooking habits, and who is responsible for preparing food in the household were the main determinants of nutrition knowledge score at *p* < 0.05. The mean score of the students was 40.06 ± 9.89 out of 84 points. Females scored significantly higher than males, at 41.10 ± 9.29 and 38.72 ± 10.48, respectively (*p* = 0.007). Students with a BMI of ≥30 achieved significantly greater scores (mean 42.30 ± 9.41) than those who were underweight, normal, or overweight (*p* < 0.001). Students who stated that they always cook their own food achieved significantly greater scores (mean 43.69 ± 9.58) than those who did not (*p* = 0.025). Students who stated that they depend on the housekeeper for food preparation achieved significantly lower scores (mean 38.86 ± 10.13) than those who prepare their own food or depend on their relatives to prepare food (*p* = 0.042). Poor nutrition knowledge was found among prospective teachers studying in the College of Basic Education. This must be rectified for the effective implementation of nutrition education programs in schools.

## 1. Introduction

Poor health behaviour has previously been observed among the Kuwaiti population as a result of rapid modernization which has led to a nutrition transition [1–3]. This puts the population at significant risk of developing overweight and obesity. According to the 2018 Kuwait Nutrition Surveillance System, there is an alarmingly high prevalence of being overweight and obese among the Kuwaiti population. It was estimated that 79.8% of Kuwaiti adults were either overweight (36.0%) or obese (43.8%), whereas the prevalence of being overweight and obese amongst Kuwaiti children aged ˃5 years to 19 years was 21.5% and 27.5%, respectively [4]. Obesity rates not only affect individuals' health but can also cause a huge burden on the healthcare system and its resources [5–7]. There is therefore a need for a plan of action to change and address unhealthy dietary patterns and lifestyles to reduce risks associated with obesity and diet-related chronic diseases. Developing healthy eating practices and encouraging physical activity from the early stages of life has a significant impact on children's current and future health [8]. There are many determinants of food choice, one of them being nutrition knowledge. Lack of nutrition knowledge and lack of awareness of the important role of a balanced diet may contribute to poor dietary practices [9]. Some studies suggest that nutrition knowledge has the potential to improve food choices and eating habits [9–12]. In addition, recent evidence suggests that enhancing nutrition knowledge through education-based interventions has been linked to improved dietary habits and food choices [13–15].

Nutrition education is vital because it is a means of spreading nutrition knowledge and thus combatting obesity [16]. Schools provide an important opportunity for the dissemination of essential information about healthy dietary patterns and active lifestyles and also an efficient/effective means of reaching a large segment of society aged 4–18 years. Hence, schools are an ideal environment to start the process of nutrition education via nutrition education programs being implemented into the curriculum for all ages. However, this implementation is complex and has different challenges that must be considered [17]. One of these challenges is teachers' capability. The teacher should be equipped with adequate knowledge, trained to deliver accurate nutrition information to students, and sufficiently motivated, as they are the main promoters in the education process [18]. In Kuwait, a few studies have assessed nutrition knowledge among university students [15, 19, 20]. All these studies are in agreement that nutrition knowledge among the Kuwaiti population should be improved. However, to the best of our knowledge, in Kuwait, no prior studies have examined nutrition knowledge among schoolteachers or university students in education colleges that are in the process of becoming teachers (teacher education students). Estimating the level of nutrition knowledge of prospective teachers is critical, since if lacking, it can lead to a reduction in the effectiveness of nutrition education or intervention programs in schools. Furthermore, this will give an insight into the gaps in nutrition knowledge of teachers that need to be filled in order to equip them for their future roles.

This is the first study to assess the level of nutrition knowledge and the effect of selected demographic characteristics on knowledge among undergraduate students at the College of Basic Education (CBE). The CBE, in the Public Authority Applied Education and Training (PAAET), is a governmental academic institute that provides programs that allow students to earn a teaching degree in Kuwait. This study will help to fill in the gaps in the literature and establish the foundational need for creating intervention programs tailored to the needs of teachers/teacher education students. Therefore, the aim of the current study has been to evaluate the level of general nutrition knowledge and demographic variations in knowledge in a sample of students attending the CBE.

## 2. Materials and Methods

### 2.1. Research Design and Study Population

A cross-sectional study was carried out among undergraduate students studying at the CBE in Kuwait from September 2019 to October 2019. The study was reviewed and approved by the Scientific Ethics Committee at the Public Authority for Applied Education and Training, Kuwait PAAET, Kuwait (number 426/2019). The study was conducted according to the guidelines laid down in the Declaration of Helsinki, and all procedures involving participants were conducted after obtaining agreement.

A variety of classes from different departments were invited to participate in the study. Permission from the professors who showed interest in the study was obtained, and the questionnaire was arranged to be applied at the professors' convenience. The questionnaire was applied in the classroom, with the presence of one of the researchers at all times to answer questions, and under the supervision of the professor. Students were given full details of the study protocol and were informed that participation was anonymous, voluntary, and would not affect their grades. Moreover, students were informed that they could withdraw from the study at any time and that answering truthfully was paramount to making the study successful. Respondents excluded from the study were those students who were absent on the day of data collection and those who did not complete the questionnaire appropriately. Informed consent was obtained from all participants.

According to the admission and registration office at CBE, the total population of students is about 25,000 [21]. Therefore, the minimum recommended sample size was 379 students, with a confidence level of 95%, a margin of error of 5%, and a response distribution of 50% using an automated Raosoft sample size calculator [22]. This software is highly recommended for this type of study [23, 24]. The sample exceeded and covered the recommended minimum sample size.

### 2.2. Development of the Questionnaire

The nutrition knowledge questionnaire was adapted and modified from a recently validated version of the General Nutrition Knowledge Questionnaire revised version (GNKQ-R) for UK adults [25], originally developed by Parmenter and Wardle in 1999 [26]. The questionnaire was translated into Arabic and back-translated into English by a certified translator and then reviewed by nutrition professionals to assess the accuracy and formulation of each question in both the English and Arabic versions. Moreover, the questionnaire was then subjected to an evaluation amongst three experts in the same field to identify any ambiguities, any lack of clarity, or any gaps in the questionnaire. The questionnaire was amended where appropriate to address all comments. Some changes had to be made to the questionnaire due to the dietary restrictions imposed by religion and cultural dietary habits. It was important for the research team to adapt the questions to reflect the specific dietary eating habits of Kuwaitis. For example, references to pork and alcohol were removed. In addition, British foods uncommon to Kuwaiti food culture were replaced with more applicable yet similar alternatives (for example, baked beans were replaced by chickpeas).

The questionnaire consisted of three main sections. The first section included basic demographic and academic information about students such as age, sex, nationality, which governorate they live in, marital status, mother's employment status, student's year of study, and academic major. In addition, the students were asked to record their height and weight.

The second section consisted of 84 items that covered four areas of nutrition knowledge presented in the following order: part 1 covered awareness of dietary recommendations (seventeen items), part 2 covered sources of nutrients (thirty-six items), part 3 covered everyday healthy food choices (ten items), and part 4 covered diet-disease relationship (twenty-one items).

The final section was added to the original questionnaire and consisted of questions chosen to explore a possible relationship between certain factors and the objective of the study. The questions investigated the primary and preferred source for obtaining nutrition advice or information, how often the student cooks his/her own food, and who usually prepares and cooks food at home.

For pilot research, the questionnaire was distributed amongst a group (*n* = 60) of students similar to the target study group. They were asked to complete the questionnaire and to give their comments regarding the clarity, feasibility, and duration of the questionnaire. Further modifications were made based on the students' comments. The results of the pilot were not included in the data analysis. The average completion time of the questionnaire was 20–25 minutes.

### 2.3. Anthropometry

Self-reported height and weight were collected through the questionnaire. Body mass index (BMI) was calculated as body weight in kilograms divided by the square of the height in meters (kg/m^2^). Weight status was then classified into four categories in accordance with the World Health Organization (WHO) categories: underweight (BMI ≤ 18.5 kg/m^2^), normal weight (BMI between 18.5 and 24.9 kg/m^2^), overweight (BMI between 25 and 29.9 kg/m^2^), and obese (BMI ≥ 30 kg/m^2^) [27].

### 2.4. Response Coding and Item Categorization

Raw data from each participant's responses were coded numerically. The score assigned for every correct answer was 1 point; otherwise, 0 points were given; then, the achievement score was computed for each part of section 2 and for section 2 as a whole to give an overall knowledge score out of 84. A higher score reflected greater knowledge. In addition, each student's overall knowledge was transformed into a percentage and then categorized into three levels using original Bloom's cutoff points [28]. The score of knowledge varied from 0 to 84 points; if the score was between 80 and 100% (67–84 points), it was considered a high level, if the score was between 60 and 79% (50–66 points), it was considered a moderate level, and if the score was less than 60% (>50 points) it was considered a poor level.

### 2.5. Statistical Analysis

Statistical analysis software (IBM Corp. Released 2010. IBM SPSS Statistics for Windows, version 23.0, Armonk, NY: IBM Corp) was used [29]. Upon completion of data entry into the SPSS PASW statistical software and before starting the analysis, it was essential to check the data for errors. Data cleaning took place by checking the outliers that may occur due to typing mistakes. An internal reliability test was conducted using Cronbach's alpha for each section and for the total scale of the questionnaire. Using this approach of Cronbach's alpha (*α*), ˃0.7 represented a minimum requirement acceptable for internal consistency of the instrument [30].

The overall internal reliability based on 84 items in the current study was high (Cronbach's alpha = 0.91), larger than the threshold of 0.7. Section 2 parts 1 and 3 had a Cronbach's alpha of 0.64 and 0.63, respectively, which is lower than the threshold. Parts 2 and 4 had an alpha value larger than the threshold (see Table 1).

Total and section scores were assessed for normality by inspection of the normal probability plots and by using the Shapiro–Wilk test. The results showed that the data were normally distributed. Descriptive analysis was carried out to provide a profile of the sample (demographic characteristics) and presented as a mean and standard deviation (±SD) for continuous variables and a frequency and percentage (%) for categorical variables. Univariate analysis was used to examine the effect of demographic characteristics on nutrition knowledge scores. An independent sample *t*-test was used for binary comparisons (nutrition knowledge between sex and nationality) and analysis of variance (ANOVA) was utilized for multiple comparisons (to evaluate the nutrition knowledge with the other demographics parameters). Stepwise multiple regression (bidirectional elimination) was used to explain the variance in nutrition knowledge scores within the sample. Statistical results were significant at *p* < 0.05.

## 3. Results

### 3.1. Study Sample Characteristics

A total of six hundred and twenty undergraduate students at the CBE participated in this study. However, twenty-three individuals were excluded (*n* = 23, 3.7%). A response rate of 96% (*n* = 597) was achieved (44% male and 56% female), which covered the recommended sample size.

The basic characteristics of the participants are shown in Table 2. The age of students ranged between 17 and 47 years, with a mean age of 21.3 ± 3.9 years (the mean ages for males and females were 21.5 ± 3.9 years and 21.2 ± 3.9 years, respectively). The majority of respondents were Kuwaiti (90%). Of the participating students, 80.9% reported being single. Only 9.2% reported having children. The mean BMI was 25.5 ± 5.9 kg/m^2^, which is indicative of the overweight category according to the definition of the WHO (males 26.1 ± 6.7 kg/m^2^; females 25.2 ± 5.1 kg/m^2^).

### 3.2. Level of Nutrition Knowledge Based on Original Bloom's Cutoff Point

Overall, the majority of respondents had a poor level of nutrition knowledge (84.1%) and there was no significant difference between the sexes using original Bloom's cutoff points (*p* = 0.096). Only 15.9% of respondents had a moderate level of knowledge. Although the percentage of females with a moderate level of knowledge (18.1%) was higher than that of males (13.1%), the difference was minute. Moreover, it was found that none of the students fell into the high categories (see Table 3).

### 3.3. Demographic Variation in Nutrition Knowledge Using Univariate Analysis

The demographic variation in nutrition knowledge among students is presented in Table 4. Out of a maximum score of 84, the mean score of the students was 40.06 ± 9.89. Females scored significantly higher than males, at 41.10 ± 9.29 and 38.72 ± 10.48, respectively (*p* = 0.004). Significant differences were observed in nutrition knowledge score among students with reference to their BMI categories. Students with BMI ≥30 achieved significantly greater score (mean 42.30 ± 9.41) in nutritional knowledge than those who were underweight, normal, or overweight (*p* = 0.001).

With regard to the major subject of study, significant differences in the knowledge score were found between students of different majors (*p* = 0.011). Science and technology students had the highest mean score of knowledge at 42.10 ± 9.65. Surprisingly, physical education and sport students had the lowest mean score at 36.82 ± 10.95. There was no statistical difference found between other demographic variables and the knowledge score.

This study found that those students who stated that they always cook their own food achieved a significantly greater mean score of 43.69 ± 9.58 in nutrition knowledge than those who did not (*p* ≤ 0.001), with the lowest mean nutrition knowledge of 34.20 ± 11.82 being achieved by those who stated that they never cook their own food. In addition, it is interesting to note that students who stated that they depend on a housekeeper to prepare food for their family achieved significantly lower score (mean 38.86 ± 10.13) in nutrition knowledge than those who prepare their own food by themselves (mean 44.79 ± 9.52) or depend on their relatives, especially mothers or wives (mean 40.79 ± 9.52) (*p* = 0.001).

### 3.4. Descriptive Statistics of Section (3) in the Questionnaire

**Figure 1:**
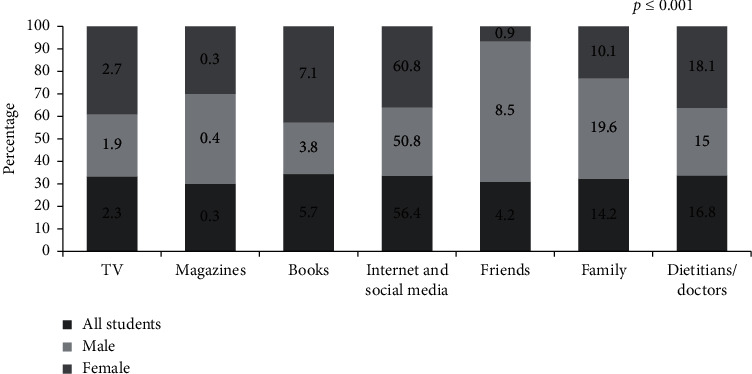
Sources of nutrition knowledge information among study participants.

Among the sources of information (Figure 1), the Internet and social media were found to be the major sources of nutrition information for the students (56.4%), whereas magazines (0.3%) were the least frequently chosen. Although both males and females shared the Internet and social media as their primary source of nutritional information, there were highly significant differences in their use of other sources (*p* ≤ 0.001). The second major source of nutrition information for males was family (19.6%), whereas for females it was dietitians and doctors (18.1%).

**Figure 2:**
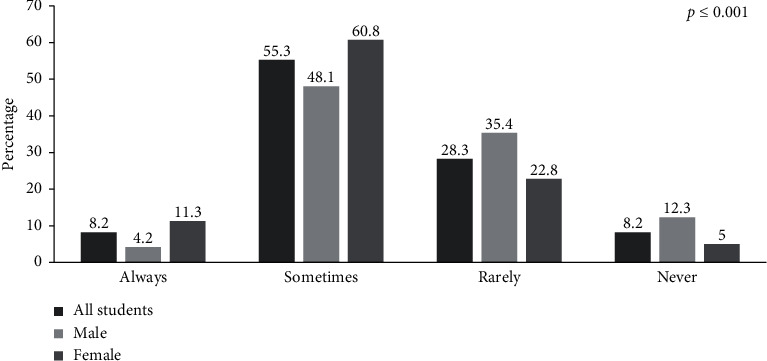
Cooking habits among study participants.

With regard to cooking habits (Figure 2), over half (55.3%) of students stated that they “sometimes” cook their own food; only a small group (8.2%) said that they “always” or “never” cook their own food. Highly significant differences were found between the sexes (*p* ≤ 0.001).

**Figure 3:**
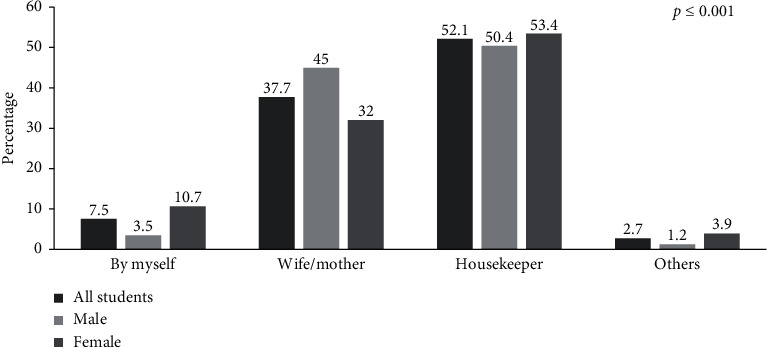
Who is responsible for preparing food in the household among study participants.

As shown in Figure 3, 52.1% of students indicated that a housekeeper was the main person responsible for preparing food for the family, followed by a wife or mother (37.7%), with a statistically significant difference between sexes (*p* ≤ 0.001).

### 3.5. Multivariate Analysis

In order to draw final conclusions regarding the effects of each variable on nutrition knowledge score, and since univariate comparisons may possibly be affected by the presence of confounding variables that are not included in the comparison each time, multiple linear regression analysis was used. The significant predictors from the univariate analysis were entered into the model. The results of the model were significant, *F* (4, 559) = 10.27, *p* < 0.001, *R*^2^ = 0.07, indicating that approximately 7% of the variance in total nutrition knowledge scores is explainable by cooking habits, BMI, sexes, and who is responsible for preparing food in the household (Table 5). It is interesting to note that “major subject of study” was not statistically significant in this model whereas it was considered significant in the univariate analysis.

## 4. Discussion

In order to investigate the general nutrition knowledge of CBE students, GNKQ-R for UK adults was adapted and modified, since currently there is no validated tool in Kuwait. This questionnaire was selected because it has been reported as being a valid tool amongst UK adults [25]. Furthermore, it has also been adopted and validated amongst other populations [31–39]. An internal reliability test was performed for each section and the overall questionnaire; the results were adequate and comparable to previous studies, as presented in Table 1. The results showed low internal reliability for the section on dietary recommendations and the section on everyday healthy food choices, with Cronbach's alpha = 0.64 and 0.63, respectively, which is lower than the threshold. These results are in line with reported values [31–33]. Other studies sharing similar results have attributed them to the differing educational backgrounds of participants due to their heterogeneity, and divergent understandings of nutrition messages due to an absence of unified dietary guidelines and insufficient nutrition education [33, 38, 40]. Some authors have stated that a reliability coefficient of 0.63 could be acceptable [36].

This study set out to investigate the nutrition knowledge of prospective teachers at the CBE in Kuwait for the first time. The results of this study indicate that the overall level of nutrition knowledge of the students is minimal; 84.1% of the students had a poor level of general nutrition knowledge using Bloom's cutoff points. Only 15.9% of respondents had a moderate level. The findings of the current study are consistent with those of Al-Isa and Alfaddagh [20], who reported a low level of nutrition knowledge among Kuwaiti male students in different years of study at Kuwait University. On the other hand, El-Sabban and Badr [19] found that most first-year students at Kuwait University (64%) had a fair level of nutrition knowledge, and around 22% of them had a poor level of nutrition knowledge, with only 14% of them having a good level. This can be explained by the methodological differences in assessing nutrition knowledge. All previous studies carried out in Kuwait and the current study agree on the necessity of improving students' nutrition knowledge.

Moreover, this study investigated various demographic characteristics and factors that may affect the nutrition knowledge score of students. Sex, BMI, cooking habits, and who is responsible for preparing food in the household were the main determinants of the nutrition knowledge score at *p* < 0.05. Although both males and females had a poor level of knowledge, the females achieved a higher mean score of knowledge (41.10 ± 9.29) than the males (38.72 ± 10.48). Similar findings were observed in previous studies [31, 33, 41, 42]. It is not surprising that female students know more about food and nutrition than males, since they are more likely to be concerned about their body image and weight [42, 43]. They also tend to diet and use weight loss strategies more frequently than males [44, 45]. In addition, females seek nutritional counselling from professionals more frequently than males [46]. In this study, females were more likely to source their nutrition knowledge from professionals such as dietitians and doctors than males. Furthermore, female students reported more often than males that they cooked on a daily basis (always) and prepared their own meals. This could be another possible explanation for the higher knowledge score achieved by females. In addition, an explanation could be that females in Kuwait are taught the subject of home economics at public schools while males are not. Home economic education provides nutrition knowledge, cooking skills, and theoretical and practical experiences regarding how to plan and prepare healthy meals. Even though female students are taught this subject, their level of nutritional knowledge is still poor. This could be due to a lack of interest in the subject of home economics. This subject is given low priority by students, parents, and the school administration in comparison with more core subjects such as science, math, and languages. Prior studies by Oogarah‐Pratap et al. [47] and Worsley et al. [48] found that home economic education is associated with better food and cooking skills, especially among boys, and it is a major source of nutrition knowledge. Therefore, it is important to introduce the subject of home economics in the education system for both sexes and to give the subject greater importance in order to increase the nutrition awareness and knowledge of students in Kuwaiti schools. Finally, the fact that there were more females than males who majored in science and technology-related fields within the study sample could be a further explanation of the result.

Another finding was that the highest score for general nutrition knowledge was obtained among obese students, compared to those students underweight, normal, and overweight. This finding is consistent with that of Labban [49], who found the highest nutritional knowledge score among Syrian students with a BMI >30. This result may be explained by the fact that obese individuals often seek help regarding nutritional guidance and advice from professionals to lose weight. It is important to acknowledge that overweight and obese individuals may have good nutrition knowledge but do not always make use of it in terms of making healthy food choices [50]. The results of the current study are different to those of O'Brien and Davies [51] and Zhou et al. [52], who observed a comparable level of nutrition knowledge among obese and nonobese individuals.

The findings of the current study suggest that cooking habits and who is responsible for preparing food in the household were the main determinants of the nutrition knowledge score at *p* < 0.05. Students who stated that they “always” cook their own food achieved significantly greater scores in nutrition knowledge than those who stated that they “sometimes,” “rarely,” or “never” cook. In addition, students who prepare their own food achieved a greater mean score in nutrition knowledge than those who do not, especially those who depend on their housekeeper for food preparation. It may be that these students are more interested in nutrition or in food and wellbeing, which manifests in their keenness to prepare their own food. However, although their nutrition knowledge is relatively higher, it is still poor. This could indicate that their source of nutrition information may be misleading. Another explanation, as shown by the results, is that a higher proportion of females than males reported “always” cooking and that they cook their own meals, and as mentioned earlier, females had higher mean nutrition knowledge than males. To develop a fuller picture, further studies on the current topic are recommended.

Contrary to expectations, physical education and sport students had the lowest nutrition knowledge score, though it was not significantly different to other students in the regression model. This finding was also reported by Ozdoğan and Ozcelik [53], who found low nutrition knowledge among students studying in universities to become coaches and physical education teachers. This finding suggests that there is a lack of adequate nutrition information in physical education and sport students' curriculums at CBE and that a change in their curriculum to address this issue may be necessary. It is important that coaches and physical education teachers have adequate knowledge in nutrition.

### 4.1. Strengths and Limitations

The strength of this study is mainly represented by the attention given to an important but neglected population in surveys on nutrition knowledge in Kuwait that may represent a risk factor in terms of nutrition education and interventions in schools.

This study has some limitations. Due to time and resource constraints, the study focused only on assessing the nutrition knowledge of the students and not their daily food intake. Therefore, it was not possible to establish a solid relationship between nutrition knowledge and eating patterns as social phenomena. In addition, this limited the capability of confirming the suggested theory of some studies that nutrition knowledge has the potential to improve food choices and eating habits. Longitudinal studies are needed to further investigate this theory.

One of the methodological limitations in this study was the use of a cross-sectional study design which had a nonrandom sample. Therefore, the generalizability of findings to other students in other institutions is limited. Moreover, accessing all the classes in the college was impossible, and the recruitment was restricted to classes where permission was granted by the respective professors to distribute the questionnaire. Since the data collection instrument was a structured and self-reported questionnaire, the answers might be affected by students' feeling at the time they filled out the questionnaire. Furthermore, the length of the questionnaire which takes around 20 minutes to complete could be a negative factor since it may have caused some burden to the participants.

Although the study questionnaire was adapted and modified from a recently validated version of GNKQ-R for UK adults and is validated amongst other populations, it has not yet been validated among a Kuwaiti population, which could add to the limitations in the statistical analysis and the power of the significance (even if the overall internal reliability of the questionnaire was adequate in this study). Finally, the use of self-reported data for height and weight can possibly cause measurement bias.

## 5. Conclusion

Poor nutrition knowledge was found among prospective teachers studying in the CBE. Teachers are an important element in the educational process; they are a powerful factor that can influence the nutrition education of children and can make changes in children's dietary behavior to prevent obesity-related diseases later in life. Therefore, teachers should have sufficient skills and nutrition knowledge. To achieve that, CBE students need to be targeted for nutrition education so that they may effectively assist in the future implementation of nutrition education programs in schools. Moreover, additional studies should be undertaken that consider actual dietary intake, attitudes, and beliefs and investigate the relationship of these factors to nutrition knowledge.

## Figures and Tables

**Table 1:** A comparison of the internal reliability of the current study with the results of questionnaires developed in other studies.

Knowledge section (2) (max score for the current study)	Current study *n* = 597	UK sample [26] *n* = 168	UK sample [25] *n* = 266	Australian sample [31] *n* = 156	Turkish sample [32] *n* = 195	Romanian sample [33] *n* = 412
P1	0.64	0.70	0.70	0.53	0.47	0.53
P2	0.87	0.95	0.86	0.88	0.88	0.82
P3	0.63	0.76	0.72	0.43	0.43	0.53
P4	0.76	0.94	0.77	0.81	0.81	0.72
Total^*∗*^	0.91	0.97	0.93	0.92	0.89	0.88

P1: dietary recommendations, maximum score = 17; P2: sources of nutrients, maximum score = 36; P3: everyday healthy food choices, maximum score = 10; P4: diet-disease relationship, maximum score = 21. ^*∗*^Total nutrition knowledge = 84.

**Table 2:** Sociodemographic characteristics of the study participants.

Students' characteristics		All, *n* (%)	Males, *n* (%)	Females, *n* (%)	*p* value
Nationality	Kuwaiti	597 (100)	260 (43.6%)	337 (56.4%)	0.560
		537 (89.9)	238 (91.5)	299 (88.7)	
	Non-Kuwaiti	60 (10.1)	22 (8.5)	38 (11.3)	

Age	Age equal or less than 21	416 (69.8)	157 (60.4)	259 (76.9)	0.116
	Age 22–27	146 (24.5)	92 (35.4)	54 (16.0)	
	Age equal or more 28	34 (5.7)	11 (4.2)	23 (6.8)	

Governorate	Al Asemah	80 (13.4)	45 (17.3)	35 (10.4)	0.086
	Hawalli	92 (15.4)	46 (17.7)	46 (13.6)	
	Al Farwaniya	132 (22.1)	65 (25.0)	67 (19.9)	
	Al Ahmadi	133 (22.3)	32 (12.3)	101 (30.0)	
	Al Jahra	105 (17.6)	52 (20.0)	53 (15.7)	
	Mubarak Al-Kabeer	55 (9.2)	20 (7.7)	35 (10.4)	

Major subject of study	Science and Technology	119 (20.6)	28 (10.8)	91 (28.6)	<0.001
	Humanities and Social Science Educational Technology Library and Information Science	225 (38.9)	110 (42.3)	115 (36.2)	
	Physical Education and Sport	56 (9.7)	46 (17.7)	10 (3.1)	
	Applied Arts and Music	178 (30.8)	76 (29.2)	102 (32.1)	

Year of study	First year	133 (22.4)	87 (33.5)	46 (13.6)	0.001
	Second year	210 (35.4)	30 (11.5)	180 (53.4)	
	Third year	148 (25.0)	75 (28.8)	73 (21.7)	
	Four or more	102 (17.2)	68 (26.2)	34 (10.2)	

Marital status	Single	483 (80.9)	240 (92.3)	243 (72.1)	<0.001
	Married/divorced	114 (19.1)	20 (7.7)	94 (27.9)	

Have children	No	542 (90.8)	250 (96.2)	292 (86.6)	<0.001
	Yes	55 (9.2)	10 (3.8)	45 (13.4)	

No. of children (*n* = 55)	1-2 children	32 (58.2)	4 (40.0)	28 (62.2)	0.198
	3-4 or more	23 (41.8)	6 (60.0)	17 (37.8)	

Mother employment	Housewife	241 (40.4)	94 (36.2)	147 (43.6)	0.313
	Works	218 (36.5)	97 (37.3)	121 (35.9)	
	Retired	138 (23.1)	69 (26.5)	69 (20.5)	

BMI	Underweight	46 (7.9)	25 (9.6)	21 (6.2)	0.001
	Normal weight	266 (45.6)	114 (43.8)	152 (45.1)	
	Overweight	151 (25.9)	49 (18.8)	102 (30.3)	
	Obese	120 (20.6)	65 (25.0)	55 (16.3)	

**Table 3:** Nutrition knowledge of students (section 2 in the questionnaire) based on original Bloom's cutoff point.

Knowledge scores	Bloom's cutoff					
Knowledge section (2)	Min	Max	Mean ± SD	Poor, *n* (%)	Moderate, *n* (%)	High, *n* (%)
P1	1	14	8.42 ± 2.44	473 (79.2)	118 (19.8)	6 (1.0)
P2	0	30	17.25 ± 5.41	460 (77.1)	132 (22.1)	5 (0.8)
P3	0	10	4.86 ± 1.93	379 (63.5)	168 (28.1)	50 (8.4)
P4	0	17	9.53 ± 3.07	500 (83.8)	94 (15.7)	3 (0.5)
Total^*∗*^	5	64	40.06 ± 9.89	502 (84.1)	95 (15.9)	0 (0.0)

P1: dietary recommendations, maximum score = 17; P2: sources of nutrients, maximum score = 36; P3: everyday healthy food choices, maximum score = 10; P4: diet-disease relationship, maximum score = 21. ^*∗*^Total nutrition knowledge = 84.

**Table 4:** Univariate analysis of demographic variance in nutrition knowledge among study participants.

Characteristics	P1	P2	P3	P4	Total knowledge^*∗*^
	Mean ± SD	Mean ± SD	Mean ± SD	Mean ± SD	Mean ± SD
*Sex*					
Male	8.18 ± 2.59	16.74 ± 5.83	4.48 ± 2.02	9.32 ± 2.98	38.72 ± 10.48
Female	8.61 ± 2.30	17.65 ± 5.03	5.15 ± 1.80	9.70 ± 3.13	41.10 ± 9.29
*p* value	0.035	0.046	<0.001	0.136	**0.004**
*Nationality*					
Kuwaiti	8.39 ± 2.45	17.23 ± 5.37	4.85 ± 1.93	9.51 ± 3.05	39.98 ± 9.85
Non-Kuwaiti	8.67 ± 2.29	17.47 ± 5.75	4.92 ± 1.91	9.72 ± 3.29	40.77 ± 10.34
*p* value	0.411	0.745	0.798	0.625	0.560
*Age*					
Less than 21	8.45 ± 2.30	17.14 ± 5.11	4.85 ± 1.87	9.37 ± 3.00	39.81 ± 9.28
From 22 to 27	8.11 ± 2.65	17.22 ± 6.23	4.82 ± 2.13	9.88 ± 3.15	40.03 ± 11.58
More than 28	9.35 ± 2.91	18.91 ± 4.96	5.09 ± 1.81	10.12 ± 3.44	43.47 ± 9.02
*p* value	0.025	0.185	0.761	0.115	0.116
*Governorate*					
Al Asemah	8.44 ± 2.42	17.89 ± 4.92	5.05 ± 1.77	9.50 ± 3.02	40.88 ± 8.62
Hawalli	8.43 ± 2.49	18.25 ± 5.35	5.10 ± 2.29	9.54 ± 3.37	41.33 ± 10.75
Al Farwaniya	8.24 ± 2.53	17.05 ± 5.47	4.57 ± 1.87	9.70 ± 2.91	39.55 ± 9.95
Al Ahmadi	8.07 ± 2.40	16.18 ± 5.43	4.83 ± 1.83	9.41 ± 2.95	38.50 ± 9.88
Al Jahra	8.83 ± 2.45	16.58 ± 5.44	4.75 ± 1.90	9.45 ± 3.17	39.61 ± 9.78
Mubarak Al-Kabeer	8.87 ± 2.13	19.02 ± 5.33	5.11 ± 1.88	9.62 ± 3.22	42.62 ± 9.83
*p* value	0.140	0.004	0.266	0.983	0.086
*Marital status*					
Single	8.35 ± 2.40	17.26 ± 5.36	4.86 ± 1.94	9.50 ± 3.07	39.96 ± 9.84
Married	8.87 ± 2.54	17.12 ± 5.77	4.88 ± 1.93	9.64 ± 3.06	40.51 ± 10.25
Divorced	6.71 ± 2.28	18.57 ± 3.04	4.43 ± 1.39	10.14 ± 3.67	39.86 ± 8.51
*p* value	0.024	0.78	0.83	0.78	0.087
*Have children*					
No	8.36 ± 2.42	17.16 ± 5.38	4.84 ± 1.92	9.49 ± 3.02	39.85 ± 9.80
Yes	9.05 ± 2.58	18.16 ± 5.70	4.98 ± 1.96	9.95 ± 3.53	42.15 ± 10.63
*p* value	0.043	0.190	0.612	0.296	0.101
*No. of children (n* *=* *55)*					
1-2 children	9.31 ± 2.42	17.56 ± 6.12	4.78 ± 2.15	10.03 ± 3.41	41.69 ± 11.69
3-4 and more	8.70 ± 2.80	19.00 ± 5.07	5.26 ± 1.68	9.83 ± 3.76	42.78 ± 9.18
*p* value	0.388	0.361	0.377	0.834	0.710
*Mother employment*					
Housewife	8.36 ± 2.44	17.26 ± 5.77	5.03 ± 1.88	9.79 ± 2.99	40.44 ± 10.24
Works	8.78 ± 2.28	17.14 ± 4.99	4.84 ± 1.96	9.61 ± 2.95	40.36 ± 9.23
Retired	7.96 ± 2.60	17.42 ± 5.42	4.58 ± 1.93	8.97 ± 3.34	38.93 ± 10.26
*p* value	0.008	0.891	0.092	0.041	0.313
*BMI*					
Underweight	7.91 ± 2.27	15.98 ± 5.82	4.46 ± 1.53	8.76 ± 3.10	37.11 ± 10.05
Normal	8.20 ± 2.38	16.85 ± 5.31	4.70 ± 1.95	9.17 ± 2.98	38.91 ± 9.47
Overweight	8.60 ± 2.27	17.64 ± 5.60	5.02 ± 2.00	10.15 ± 3.31	41.41 ± 10.44
Obese	8.91 ± 2.41	18.23 ± 5.19	5.23 ± 1.85	9.93 ± 2.79	42.30 ± 9.41
*p* value	0.019	0.033	0.024	0.002	**0.001**
*Major subject of study*					
Science and Technology	8.74 ± 2.33	17.89 ± 5.41	5.17 ± 1.85	10.30 ± 3.08	42.10 ± 9.65
Humanities and Social Science Educational Technology Library and Information Science	8.41 ± 2.21	17.26 ± 5.14	4.64 ± 1.87	9.52 ± 3.00	39.83 ± 9.21
Physical Education and Sport	7.88 ± 2.47	15.66 ± 5.80	4.57 ± 2.16	8.71 ± 3.04	36.82 ± 10.95
Applied Arts and Music	8.44 ± 2.73	17.51 ± 5.63	5.01 ± 1.97	9.29 ± 3.14	40.24 ± 10.53
*p* value	0.185	0.079	0.043	0.006	**0.011**
*Year of study*					
First year	8.20 ± 2.56	17.32 ± 4.81	4.73 ± 1.93	9.61 ± 2.83	39.85 ± 9.03
Second year	8.66 ± 2.33	17.56 ± 5.17	5.18 ± 1.79	9.67 ± 3.04	41.06 ± 9.27
Third year	8.40 ± 2.44	17.32 ± 5.88	4.63 ± 1.96	9.26 ± 3.19	39.60 ± 10.48
Fourth or more	8.22 ± 2.51	16.52 ± 5.88	4.68 ± 2.03	9.56 ± 3.23	38.97 ± 11.11
*p* value	0.284	0.463	0.023*∗*	0.641	0.287
*Source of information*					
TV	8.71 ± 1.54	15.93 ± 3.99	4.21 ± 2.04	7.43 ± 3.48	36.29 ± 8.51
Books and magazines	8.31 ± 2.33	18.31 ± 4.72	4.86 ± 2.08	9.06 ± 3.26	40.53 ± 9.93
Internet	8.49 ± 2.38	17.30 ± 5.47	4.95 ± 1.87	9.92 ± 3.10	40.66 ± 9.90
Friends	8.20 ± 1.84	17.00 ± 5.45	4.92 ± 2.53	9.32 ± 3.01	39.44 ± 9.64
Family	7.84 ± 2.73	16.45 ± 6.01	4.44 ± 2.07	8.73 ± 3.09	37.45 ± 10.80
Dietitians/doctors	8.75 ± 2.60	17.63 ± 5.05	4.96 ± 1.72	9.44 ± 2.57	40.78 ± 8.98
*p* value	0.190	0.461	0.241	0.002	0.077
*Cooking habits*					
Always	9.14 ± 2.33	19.27 ± 4.92	5.39 ± 1.84	9.90 ± 3.63	43.69 ± 9.58
Sometimes	8.47 ± 2.37	17.38 ± 5.30	4.94 ± 1.87	9.71 ± 2.93	40.51 ± 9.66
Rarely	8.35 ± 2.35	17.12 ± 5.14	4.81 ± 1.92	9.55 ± 3.03	39.83 ± 9.16
Never	7.59 ± 3.05	14.80 ± 6.58	3.90 ± 2.13	7.92 ± 3.14	34.20 ± 11.82
*p* value	0.015	0.001	0.001	0.001	**<0.001**
*Main person who prepared food*					
By myself	9.53 ± 2.11	19.62 ± 4.41	5.47 ± 1.92	10.11 ± 3.32	44.73 ± 8.82
Wife/mother	8.51 ± 2.23	17.94 ± 5.24	4.77 ± 1.87	9.57 ± 3.12	40.79 ± 9.52
Housekeeper	8.17 ± 2.61	16.40 ± 5.49	4.84 ± 1.97	9.46 ± 2.98	38.86 ± 10.13
Others	8.94 ± 1.69	17.50 ± 5.75	4.69 ± 1.62	8.75 ± 3.49	39.88 ± 9.00
*p* value	0.003	<0.001	0.165	0.417	**0.001**

P1: dietary recommendations, maximum score = 17; P2: sources of nutrients, maximum score = 36; P3: everyday healthy food choices, maximum score = 10; P4: diet-disease relationship, maximum score = 21. *∗*Total nutrition knowledge = 84.

**Table 5:** Multiple linear regression of nutrition knowledge on sex, BMI, cooking habits, and who is responsible for preparing food in the household.

Variable	Unstandardised *β*	SE	95% CI	Standardised *β*	*T*	*p* value
(Intercept)	39.31	2.64	[34.12, 44.50]	0.00	14.89	<0.001
Cooking habits	−1.40	0.60	[−2.58, −0.22]	−0.11	−2.33	0.020
BMI category	1.68	0.45	[0.80, 2.56]	0.15	3.74	<0.001
Sex	2.06	0.84	[0.40, 3.72]	0.10	2.44	0.015
Preparing food in the household	−1.38	0.65	[−2.65, −0.10]	−0.09	−2.13	0.034

Note. Results: *F* (4, 559) = 10.27, *p* < 0.001, *R*^2^ = 0.07. Unstandardized regression equation: total all right = 39.31 − 1.40 ∗ cooking habits + 1.68 ∗ BMI category + 2.06 ∗ sex − 1.38∗who is preparing food at household. *β*: beta coefficient; SE: standard error; CI: confidence interval.

## Data Availability

The data that support the findings of this study are available from the corresponding author (W.H) upon reasonable request.

## Abbreviations

CBE: College of Basic Education
PAAET: Public Authority Applied Education and Training
BMI: Body mass index
WHO: World Health Organization.
